# Innovative Non-Surgical Plastic Technique for Saddle Nose Correction: A Study on 97 Patients

**DOI:** 10.3390/jcm13082387

**Published:** 2024-04-19

**Authors:** Riccardo Ossanna, Sara Ghazanfar Tehrani, Alessia Dallatana, Sima Khabouri, Sheila Veronese, Andrea Sbarbati, Mario Goisis

**Affiliations:** 1 Department of Neuroscience, Biomedicine, and Movement, Section of Anatomy and Histology, University of Verona, 37134 Verona, Italy; sara.ghazanfartehrani@univr.it (S.G.T.); sheila.veronese@univr.it (S.V.); andrea.sbarbati@univr.it (A.S.); 2 Department of Surgical Sciences, Dentistry, Gynecology and Pediatrics, University of Verona, 37134 Verona, Italy; alessia.dallatana@univr.it; 3 De Clinic, Viale Regina Giovanna 39, 20129 Milan, Italy; simuly_kh7@yahoo.com (S.K.); mariogoisis@yahoo.it (M.G.)

**Keywords:** saddle nose, fat-derived products, mesenchymal stem cells, hyaluronic acid, acoustic wave therapy, aesthetic enhancement

## Abstract

**Background:** Non-surgical rhinoplasty is one of the best choices in mild cases of the saddle nose, and it represents a solution for the aesthetical amelioration of facial deformity; nevertheless, in most critical cases, surgical intervention is still required. This study reports the experience and results of a single facial plastic surgeon (M.G.) using a non-surgical technique for the correction of saddle noses in a large cohort of patients. **Methods:** This retrospective study assesses all patients injected from January 2017 through October 2023 in private clinics in Milan (Italy), London (UK), and Dubai (UAE). All patients were followed up for 12 months. The harvested adipose tissues were processed with different systems and with or without acoustic wave therapy (AWT). The extracted products have been characterized in terms of cellular yield and cell growth. Ninety-seven patients were injected with adipose-derived products or hyaluronic acid (HA). Patients were followed up for 12 months, and satisfaction data were analyzed. **Results:** The stem cells obtained from the patients who previously received AWT displayed a statistically higher cell growth ability in comparison with those of the cells derived from patients who did not receive AWT. The evolution of patient satisfaction during the time for each group of treatment was investigated, and cellular treatments show the best maintenance of patient satisfaction over time. **Conclusions:** Dermgraft and AWT approaches resulted in the highest patient satisfaction for the non-surgical correction of the saddle nose deformity.

## 1. Introduction

Saddle nose represents a pathological condition caused by a loss in dorsal nasal height created by the contraction of nose cartilage, leading to the deformity of the profile. Although the hereditary condition forms are rare and usually associated with more general facial malformation syndromes, the most common cause is related to traumatic injuries. As the nasal bridge collapses following a trauma, this pathology can affect a person’s aesthetic, strongly impacting the individual from a psychological point of view. Aesthetical improvement thus represents one of the main goals in the treatment of saddle nose, with the final aim of restoring the nose profile [1,2].

Rhinoplasty is the most common surgical procedure applied for nasal reconstruction, albeit this implies common surgical-related risks. Particularly, an autologous cartilage graft is exploited to correct saddle nose deformity. Different body sites to collect cartilage in the human body are employed (septal, conchal, costal, calvarial, and iliac bones cartilage), although many limitations exist, such as the insufficient yield of material and the risk of injury in the harvest site. For these reasons, the refinement of a non-surgical correction method is acquiring huge relevance to achieve the best result in profile restoration as an alternative to standard surgical procedures [3].

Non-surgical correction is one of the best choices in mild cases of saddle nose, and it represents a solution for the aesthetical amelioration of facial deformity; nevertheless, in most critical cases, surgical intervention is still required. The non-surgical procedure consists of the injection of dermal fillers in the nasal skin–soft tissue in order to restore the nose structure, increasing the height of the nasal bridge [3]. Non-surgical corrections are characterized by many benefits, such as short recovery time, few post-surgical complications, and cost-effectiveness. Despite these positive aspects and the relatively simple procedure, the injection of the liquid solution should be carried out with caution, given the variability in vascular nasal anatomy. Less invasive techniques than surgical operations have been commonly performed since the 1800s when paraffin was exploited as a liquid dermal filler. In the following years, other solutions, such as vegetable oil and beeswax, were utilized, with related complications such as infection caused by foreign body reactions and following facial deformity. In recent years, safer fillers that are suitable to be injected into the nose have been developed [4].

Dermal fillers for cosmetic use currently approved by the US Federal Drug Administration include HA, calcium hydroxyapatite, poly-L-lactic acid and polymethyl methacrylate. The peculiar characteristic of these solutions is the low immunogenicity. Particularly, HA is a complex polysaccharide present in the human body and is thus susceptible to physiological synthesis and degradation. The advantages with respect to other dermal filler solutions are their safety and the possibility of reabsorption for the natural presence of hyaluronidase in the human body [5]. This aspect also represents a disadvantage, as it is a temporary solution, although it is helpful in the case of patient discontent after the procedure or in the presence of adverse effects. To fill this gap, researchers are currently trying to develop cross-linking methods to improve the durability of HA as filler [6]. HA is widely applied nowadays to refine different parts of the nose, not only in saddle nose correction. Non-surgical HA injections are characterized by immediate recovery time, with no swelling or pain associated, and can also be exploited after surgery operations to correct residual aesthetical defects. In fact, after the injection, the filler inside the nose is manipulated and massaged with fingers to shape, at best, the deformity [5].

Many examples in the literature report the successful application of HA as a filler that can be injected into the nose in terms of patient satisfaction and harmonization of facial traits [7,8,9]. Particularly, Baser et al. reported an increase in patient satisfaction after the non-surgical intervention, from 18 ROE (Rhinoplasty Outcome Evaluation) to 75 ROE [5], indicating a significative improvement in how the patients perceived their facial aesthetic.

HA is reported to be one of the main components of human nasal cartilage [10]. It has been demonstrated that HA can stimulate in vivo the de novo synthesis of collagen [4]. Particularly, Erisgin et al. recently proposed the use of an HA matrix as a solution for dorsal augmentation in a rabbit model. After the sacrifice, collagen quantity in nasal tissue was evaluated compared to the untreated control group; collagen was higher in the saline-gelled HA matrix-treated group [11]. Collagen is synthesized in vivo from HA filler [12]. In the literature, this is reported to be related to osteogenic differentiation [13], even if associated with complex protocols that are tedious to replicate in humans [14]. This opens the possibility to evaluate and further investigate HA for saddle nose correction, aiming also to a possible osteogenic restoration.

Fat-derived fillers represent another class of solutions frequently utilized for non-surgical nose correction. Fat can be easily collected in large amounts from the body of the patient following liposuction, representing a good candidate for autologous finality. Jeong et al. reported successful rhinoplasty utilizing fat graft for saddle nose deformity, with excellent outcomes [3]. Autologous fat is characterized by biocompatibility and is often not completely resorbed, as is common in HA fillers. Moreover, it can enhance the regenerative potential in the tissue of the injection site and surrounding tissues [4]. In recent years, fat grafting has been increasingly used in reconstructive, aesthetic, and regenerative medicine. The regenerative potential for the autologous applications of fat-derived filler resides both in the stromal vascular fraction and in the cellular compartment of the adipose tissue [15], particularly in a specific mesenchymal stem cells (MSCs) population, the adipose-derived stem cells (ADSCs). Indeed, the regenerative potential of adipose tissue is well known. Moreover, harvesting human adipose tissue by lipo-aspiration is currently considered a safe and minimally invasive procedure. Interestingly, the restoration of tissue contour and volume could be attributed to adipogenic differentiation of ADSCs. The composition of the harvested fat is a heterogeneous cell subpopulation, such as adipocytes, adipose-derived stem cells, monocytes and macrophages, extracellular matrix components, pericytes, endothelial cells, and erythrocytes. In general, this component could promote revascularization, activate local stem cell niches, modulate immune responses via paracrine secretion of numerous bioactive molecules, promote wound healing, and exhibit anti-inflammatory activity [16]. In fact, during the last few years, surgeons and scientists have studied different protocols to manipulate fat and inject the respective fat-derived product without immune-inflammatory reactions [17]. ADSCs are multipotent cells with the ability to self-renew and differentiate into multi-lineage cell subpopulations such as adipocytes, chondrocytes, or osteocytes. Moreover, the ability of ADSCs is not limited to the restoration of cell loss by direct differentiation in various cell types. They have also been employed to secrete high quantities of cytokines and growth factors (fibroblast growth factor, keratinocyte growth factor, IL-6, and IL-7, among others), which are thought to cooperate for the reparative processes in a finely tuned balance [18].

The non-surgical rhinoplasty, or liquid rhinoplasty, using fillers HA or calcium hydroxyapatite or the minimally invasive natural filler injections (Lipofilling by Microfat, Dermgraft), has become an increasingly popular alternative to classical surgical procedures [19]. Several authors published case series of corrections, mainly in case of inadequate tip projection, dorsal hump, and maximal bridge height augmentation.

This study reports the experience and results of a single clinician (M.G.) using a non-surgical rhinoplasty technique for correction of saddle nose in a large cohort of patients. To date, this is the largest case series reporting outcomes in non-surgical rhinoplasty on this specific deformity.

## 2. Materials and Methods

### 2.1. Eligibility Criteria

To be eligible for the treatment, the patient must have a depression above the supra tip of the nose not exceeding 5 mm, which can be associated with decreased projection and/or cephalic rotation of the tip (minimal and moderate saddle nose). Exclusion criteria included patients with the following characteristics: (1) a previous rhinoplasty with silicone or other alloplastic nasal implants, (2) cocaine abusers, (3) pregnancy, breastfeeding, auto-immune diseases, allergies, and (4) a marked lack of bony and cartilaginous support (major saddle nose).

### 2.2. Patients

This study assesses all patients injected from January 2017 through October 2023 by a single facial plastic surgeon (M.G.), in private clinics in Milan (Italy), London (UK), and Dubai (UAE). This study was conducted with the approval of these clinics and in accordance with the 1964 Declaration of Helsinki and its later amendments. Informed consent was obtained before any surgical procedures and for publication of the photos. Of the 97 patients treated, the age range was 19 to 71 years, with 71% aged 21 to 50 years. In total, 62% of patients had previous rhinoplasty history; in particular, 40% had undergone surgical open rhinoplasty. Forty-one patients underwent the single plane technique injection with HA filler (Genefill dx, Bioscience, Dümmer, Germany). Twenty patients underwent a Lipofilling treatment by different fat processing techniques (Coleman, Lipocube Micrograft, Seffi), and 36 patients underwent a Dermgraft and enriched Nanograft treatment. The fat, the Dermgraft, and the enriched Nanograft were harvested from the abdomen. Then, 3 weeks before the harvesting, 6 sessions of acoustic wave therapy (AWT) were performed by the Duolith device (Storz Medical AG, Tägerwilen, Switzerland) on the donor area of 32 of the 56 patients. This treatment aimed to improve the regenerative potential of the tissue.

The characteristics of the patients, the technique, complications, and satisfaction rates were noted. In particular, satisfaction was investigated immediately after the procedure, and all patients were followed up for 12 months. The satisfaction survey was repeated 3 months, 6 months, and 12 after the procedure. The satisfaction score was assigned using a questionnaire with a scale ranging from 1 to 10: 1—dissatisfied, I don’t see any result and 10—full satisfaction of the correction. Photos were taken before the injection, immediately after, and 3, 6, and 12 months later.

### 2.3. Tissue Collection

120 cc of Klein solution (2% Lidocaine solution: 0.08% *w*/*v*; Adrenaline 1 mg/mL solution: 0.1% *v*/*v* in 0.9% saline) was injected 10 min before the harvesting. A cannula of 2 mm diameter, 6 holes (Goeasy patented cannula, Go Easy system, Milan, Italy), the Coleman Cannula, and the Seffi cannula were used for harvesting. The harvested tissue was processed in Goeasy.bio system (Dermgraft, Go Easy system, Milan, Italy), Goeasy.bio enriched Nanograft (Go Easy system, Milan, Italy), Microfat (Lipocube, London, UK), Nanofat (Lipocube, London, UK), fat by the Coleman technique, and fluid fat by Seffi (Superficial Enhanced Fluid Fat Injection, Bologna, Italy). In order to evaluate the effect of AWT and the quality of fat, Dermgraft and enriched Nanograft, 24 samples were transported in an adiabatic container to the laboratory of the University of Verona. In particular, 4 samples were processed by the Coleman technique, 4 by Lipocube system/Micrograft, 4 by Lipocube system/Nanofat, 4 by Seffi, 4 by Goeasy.bio system (Dermgraft) and 4 by Goeasy.bio enriched Nanograft. Of these samples, 50% were harvested from patients who had AWT treatments, and 50% were from patients who had not.

### 2.4. Enzymatic Digestion and Culture of ADSCs

All the samples (10 mL each) were digested with collagenase type I at the concentration of 1 mg/mL (GIBCO Life Technology, Monza, Italy) dissolved in Hank’s Balanced Salt Solution (HBSS, GIBCO Life Technology, Monza, Italy) with 2% Bovine Serum Albumin (BSA, GIBCO Life Technology, Monza, Italy) for 45 min at 37 °C. Complete culture medium (Dulbecco’s Modified Eagle’s Medium (DMEM), Sigma-Aldrich, Milan, Italy), supplemented with 10% of Fetal Bovine Serum (FBS, GIBCO Life Technologies, Waltham, MA, USA), 1% 1:1 penicillin/streptomycin (P/S solution, GIBCO Life Technologies, Waltham, MA, USA) and 0.6% Amphotericin B (GIBCO Life Technologies, Waltham, MA, USA), was added to neutralize the enzyme action. After the neutralization process, the sample was centrifuged at 3000 rpm for 5 min. The cell pellet was incubated with erythrocyte lysis buffer 1× (Macs Miltenyi Biotec, Milan, Italy) for 10 min at room temperature. Again, the cell suspension was centrifuged and resuspended in a complete culture medium. Finally, the cells were filtered through a 70 µm nylon mesh. The products obtained were then cultured for the following cellular analysis.

### 2.5. Cellular Growth Capacity

The extracted ADSCs were seeded on a 25 cm^2^ T-flask with a complete culture medium and incubated in a humidified atmosphere at 37 °C with 5% CO_2_ and 20% O_2_. The first medium change was performed after 72 h from the enzymatic digestion, and the subsequent changes every 48 h. The cellular growth was performed by counting the cells after 7, 14, and 21 days of culture, and the obtained cell numbers were normalized by dividing them with the corresponding cellular yield. The number of living cells was calculated using the Trypan Blue exclusion assay in a CytoSMART counter (Automated Image-based Cell Counter, version 1.5.0.16380, CytoSMART Technologies B.V., Eindhoven, the Netherlands).

### 2.6. Statistical Analysis

Statistical analyses were performed using GraphPad Prism 7.03 for Windows (GraphPad Software, La Jolla, CA, USA). For statistical analysis, a one and two-way ANOVA test were performed, and a 95.00% confidence interval was employed to compare the evaluated groups, considering a *p*-value < 0.05 to indicate the differences that were statistically significant.

## 3. Results

### 3.1. In Vitro Results

#### 3.1.1. Cell Growth of Different Fat-Derived Products

**Figure 1:**
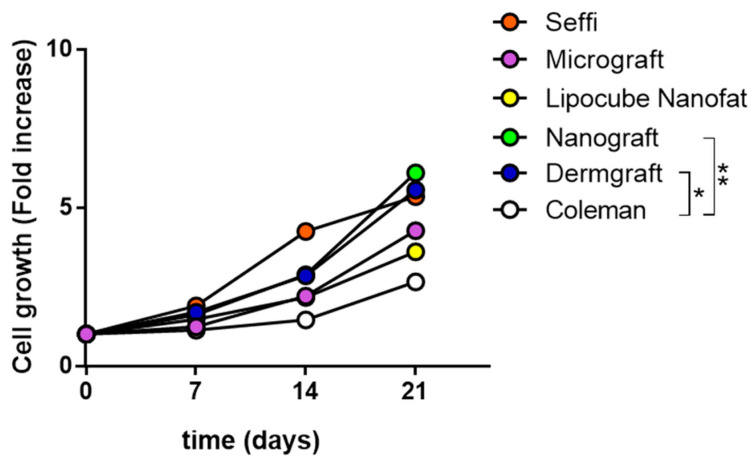
Cell growth ability of the fat-derived groups. The X axis represents the different time points, and the Y axis represents the cell growth (calculated as a fold increase). The letters are related to the different techniques and associated with different colors: Coleman technique (White), Nanograft (Green), Lipocube Nanofat (Yellow), Seffi (Superficial Enhanced Fluid Fat Injection) (Orange), Dermgraft (Dark Blue), Lipocube Micrograft (Purple). Differences between experimental conditions were analyzed with a two-way ANOVA test and post hoc Tukey post-test. Quantitative data are expressed as means ± SEM: * *p* ≤ 0.05, ** *p* ≤ 0.01.

The cell growth resulted statistically different at the last time point of growth for the stem cells obtained from the fat samples processed with the Goeasy.bio system (Dermgraft) (*p* value = 0.0345) and Goeasy.bio enriched Nanograft (*p* value = 0.0056), compared with the Coleman group. The cell growth did not show statistically differences at the last time point of growth for the stem cells obtained from Seffi, Lipocube Microfat, and Lipocube Nanofat, as shown in Figure 1.

#### 3.1.2. Cell Growth of AWT and NOT AWT

**Figure 2:**
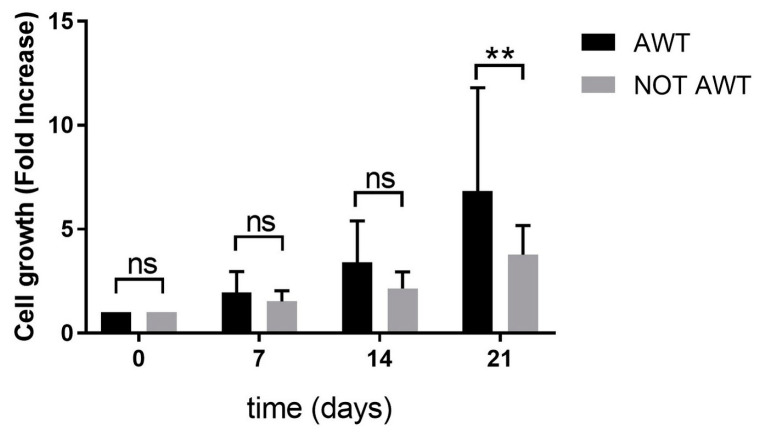
Cell growth comparison between AWT (Black) and not AWT (Grey) patients. The X axis represents the different time points, and the Y axis represents the cell growth (calculated as a fold increase). Differences between experimental conditions were analyzed with a two-way ANOVA test and post hoc Tukey post-test. Quantitative data are expressed as means ± SEM: ** *p* ≤ 0.01. “ns” refers to non-significant data (*p* > 0.05).

As shown in Figure 2, the cell growth results were statistically different at the last time point. In particular, the stem cells obtained from the patients who previously received the AWT displayed a statistically higher cell growth ability in comparison with those of the cells derived from patients who did not receive AWT (NOT AWT).

### 3.2. Clinical Results

**Figure 3:**
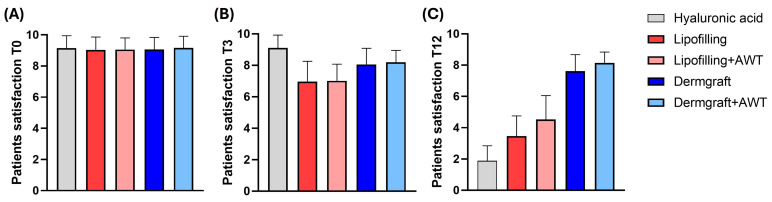
Patients’ satisfaction at 3 different time points: (**A**) T0 represents the patients’ satisfaction immediately after the treatment, (**B**) T3 after 3 months, and (**C**) T12 after 12 months. The X axis represents treatments that have been used: Hyaluronic acid (grey), Lipofilling (red), Lipofilling with AWT (pink), Dermgraft (dark blue), Dermgraft with AWT (light blue). The Y axis represents the patient’s satisfaction. Differences between experimental conditions were analyzed using the ordinary one-way ANOVA test and Tukey’s multiple comparisons test. Quantitative data are expressed as means ± SEM.

As shown in Figure 3, the graphs represent patients’ satisfaction levels with saddle nose non-surgical correction at three different time points after the treatments. T0 represents the patients’ satisfaction immediately after the treatment, T3 after 3 months, and T12 after 12 months. Graph (A) shows no significant differences between groups after 3 months of analysis. After 3 months (B), a significant difference between all the groups (****; *p* ≤ 0.0001) has been reported. Lipofilling versus Lipofilling+AWT and Dermgraft versus Dermgraft+AWT did not show a significant difference. In addition, as shown in graph (C), it is noticeable that there is a high significance between all the groups of treatments (****; *p* ≤ 0.0001), except Dermgraft versus Dermgraft+AWT, which was not statistically significant (*; *p* ≤ 0.1).

**Figure 4:**
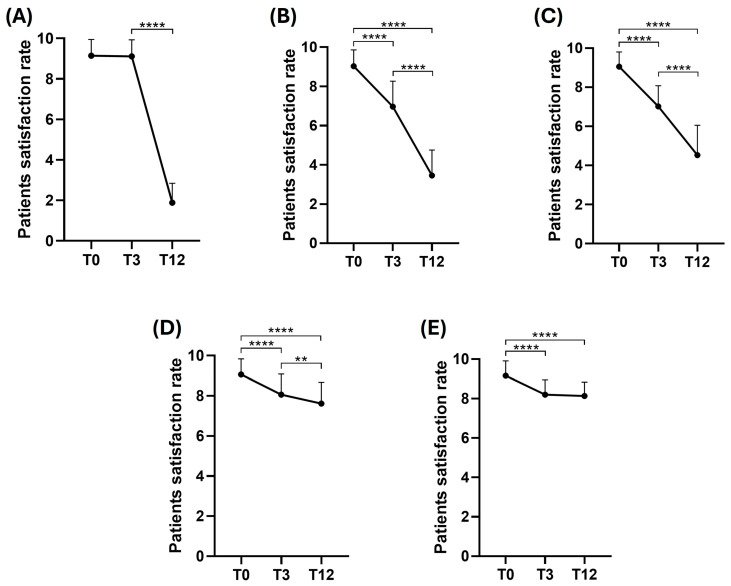
Patients’ satisfaction over the time of evaluation. Each graph represents different treatments, including (**A**) Hyaluronic acid, (**B**) Lipofilling, (**C**) Lipofilling+AWT, (**D**) Dermgraft, and (**E**) Dermgraft+AWT. The X axis represents the different time points; T0 represents the patients’ satisfaction immediately after the treatment, T3 after 3 months, and T12 after 12 months. The Y axis describes the patients’ satisfaction rate. Differences between experimental conditions were analyzed using the ordinary one-way ANOVA test and Tukey’s multiple comparisons test. Quantitative data are expressed as means ± SEM: ** *p* ≤ 0.01, **** *p* ≤ 0.0001.

Moreover, the evolution of patient satisfaction during the time for each group of treatment was investigated. As shown in Figure 4, the graphs represent the changes in patient satisfaction based on three different time points (T0, T3, and T12). T0 represents the patients’ satisfaction immediately after the treatment, T3 after 3 months, and T12 after 12 months.

Graph (A) shows a significant decrease in the HA satisfaction rate after 12 months. In this context, the slope of the graph has decreased rapidly after 3 months of treatment. Graph (B) shows a significant decrease in patient satisfaction following Lipofilling treatment after 3 months and 12 months, compared to the immediate analysis. Moreover, it shows a significant reduction after 12 months compared to T3 (****; *p* ≤ 0.0001). Graph (C) shows Lipofilling treatment with AWT that describes a significant downtrend from T0 to T12 (****; *p* ≤ 0.0001). Graph (D) shows Dermgraft treatment that describes significant differences from T0 to T3 and T12 (****; *p* ≤ 0.0001); furthermore, it shows a significant decrease between T3 to T12 (**; *p* ≤ 0.01). Graph (E) shows Dermgraft treatment with AWT, which describes a significant decrease from T0 to T3 and T12. Interestingly, the patient satisfaction of Dermgraft+AWT does not change from T3 to T12.

### 3.3. Injection Technique

**Figure 5:**
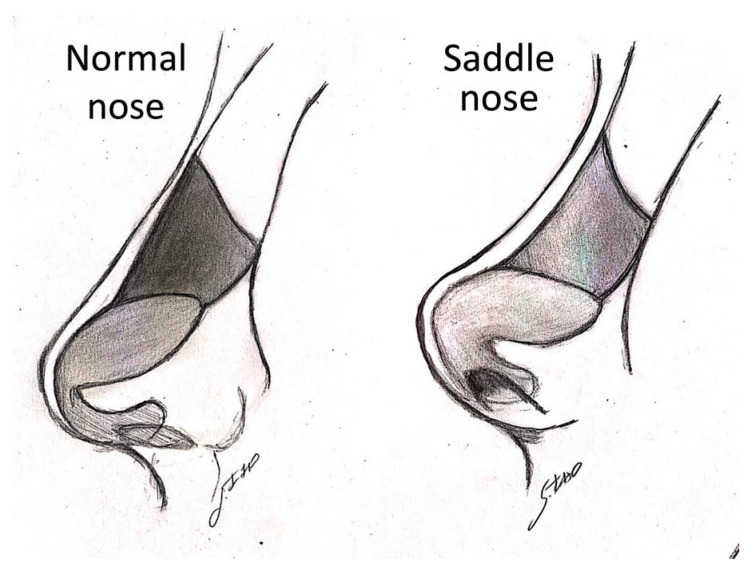
Deformity and rotation of the tip in saddle nose (courtesy of Dr. Sara Izzo).

**Figure 6:**
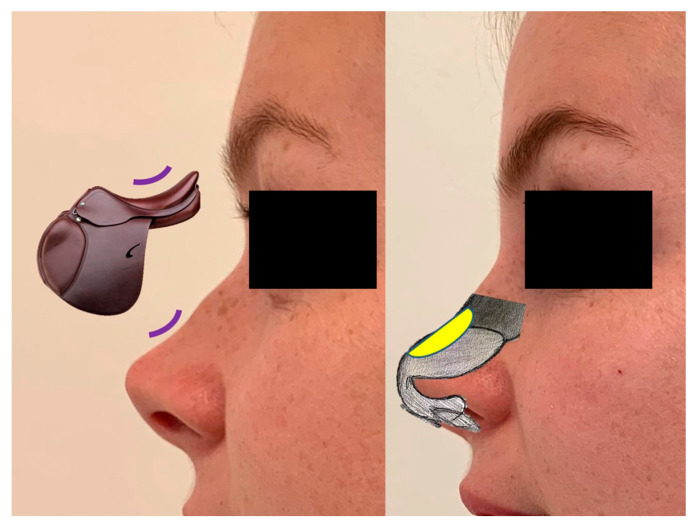
OR, 28 years old, moderate saddle nose with recession of the dorsum associated with cephalic rotation of the tip.

The technique of the author (M.G.) starts with the assessment of four critical aesthetic points on the nose: the supratip, tip, infratip, and columella. The projection, symmetry, definition, and relation between ala nasalis and columella in the normal nose and saddle nose, as reported in Figure 5, were noted. In Figure 6, a case of moderate saddle nose is schematized.

The quality and laxity of the skin are other parameters that have been tested. In particular, the laxity was assessed by a pinch test. In this contest, the evaluator’s ability to pinch skin away from the underlying bone/cartilaginous/fibrous skeleton is mandatory. In tightness of the skin envelope, where no skin pinch is possible, the correct plane of injection is likely to prove challenging to access, exposing the injector to the likely increased risks of incorrect placement, vascular injury, or injection under higher pressure. In these circumstances, treatment is avoided, and the patient is counseled about the risks.

**Figure 7:**
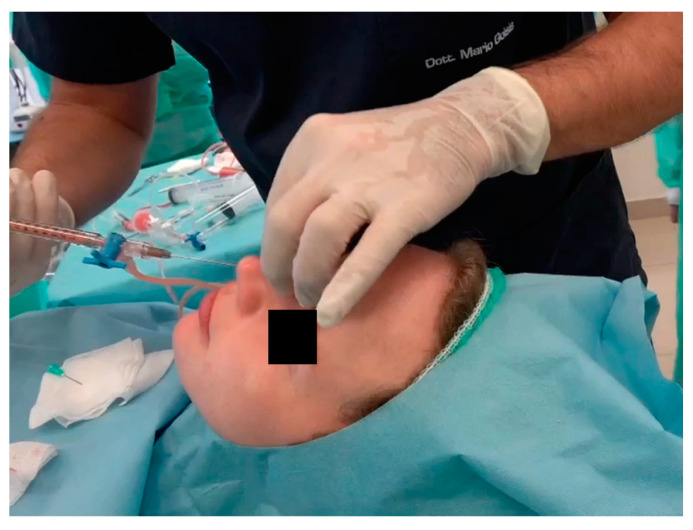
Injection of Dermgraft with a 22G 4 cm long blunt cannula.

**Figure 8:**
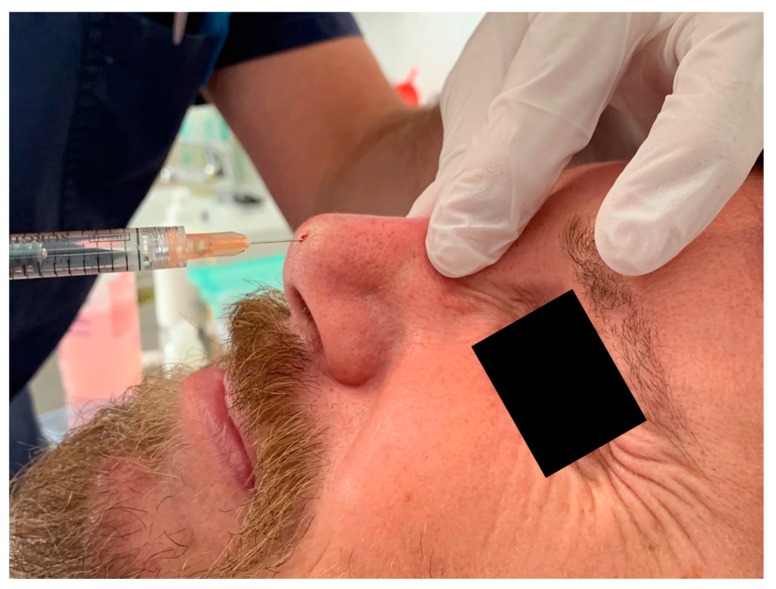
Injection of HA filler with 22G 4 cm long blunt cannula.

The injection technique involves the use of a 1 mL syringe with a 22G 4 cm long blunt cannula. The use of these cannulas enables the injector to administer a highly viscose product under low pressure. The procedure is performed retrogradely, with high-precision injections of small aliquots, as shown in Figure 7 and Figure 8.

Furthermore, the relatively large diameter of the cannula reduces the risks of intravascular injection. The other principles to ensure safety injections are to inject at the level of the midline, and to inject slowly and with low-volume injections.

The injections are carried out regularly, inserting the cannula at the level of the tip of the nose, moving it to the radix, and retracting caudally with multiple deposits of no more than 0.1 mL per injection to fill the area of the saddle. Then, the gel implant, the fat, or the Dermgraft, is massaged into place, and it can be relocated more laterally in case of lateral defects. The injection continues until the tip is reached, and the product is deposited in order to improve its projection and definition and create a supratip breakpoint.

**Figure 9:**
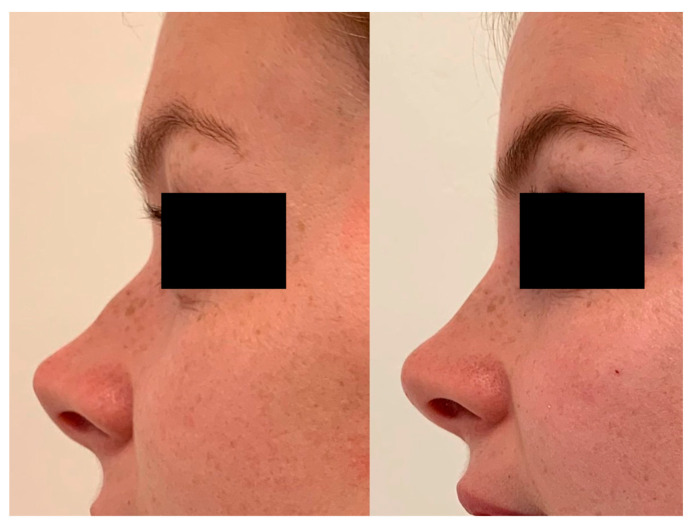
Correction of the defect 1 year after injection of Dermgraft.

**Figure 10:**
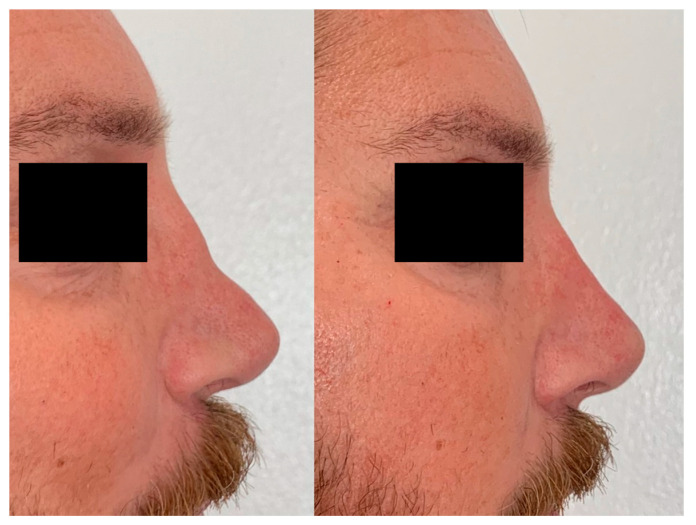
Correction of the defect 6 months after injection of HA.

In moderate saddle nose, a columellar injection can be included. In this case, the cannula is rotated 90 degrees from the same insertion point at the level of the tip of the nose, it is moved to the nasal spine, and it is retracted caudally with multiple deposits of no more than 0.1 mL per injection to fill the area of the columella. Two outcomes of the non-surgical saddle nose correction procedure are shown in Figure 9 and Figure 10.

## 4. Discussion

The cell growth results were statistically higher for the stem cells obtained from Dermgraft and Nanograft compared to the Coleman group. This could highlight that the different treatments applied to fat could influence the stem cell growth ability, which finally affects the regenerative property of the injectible products. Furthermore, the AWT effect on the different fat-derived products was studied. In this manner, the authors aimed to observe the AWT influence correlated to cell growth. As shown in the results, AWT produced a notable benefit for stem cell activity after 21 days of incubation. Both the fat manipulation procedures and the physical treatments can impact stem cell growth, and this could be translated into a higher regenerative potential.

In the second part of the study, the authors focused on the analysis of patient satisfaction over time. The results highlighted no differences in the first time point (T0) between the groups. After 3 months of analysis, patient satisfaction with HA treatment remained high in comparison to the other groups. Moreover, Lipofilling versus Lipofilling+AWT and Dermgraft versus Dermgraft+AWT did not manifest significant differences, thus suggesting that AWT treatment did not affect patient satisfaction over time. Interestingly, at the last time point (T12) of analysis, the Dermgraft and Dermgraft+AWT were the groups with the better maintenance of satisfaction after 1 year of treatment.

In addition, the authors dived deeper to study the patient’s satisfaction over time for each treatment. This study demonstrates that HA injection showed the most decreased slope after treatment. On the other hand, the cellular treatment shows preserved patient satisfaction. In particular, Dermgraft displayed the best patient satisfaction maintenance over the analysis time, compared to Lipofilling. AWT illustrated remarkable patient satisfaction maintenance above all. These results suggest that fat-derived products such as Lipofilling or Dermgraft could be used as a promising tool for nasal surgery or reconstruction more than HA. The saddle nose deformity relates to the loss of projection of the cartilaginous and/or bony structure of the dorsum of the nose. The causes of saddle nose have changed over the years: infectious and toxic causes have become less frequent, while trauma and primary or secondary reduction rhinoplasties now represent the main causes of these deformities [20].

Durbec and Disant [21] proposed a classification of the various stages of saddle nose, presented as follows:

- The minimal saddle nose corresponds to a depression above the supratip of the nose due to loss of septal support associated with slight retraction of the base of the columella, while tip projection and rotation are not affected.
- The moderate saddle nose corresponds to a more marked recession of the dorsum but not exceeding 5 mm, which can be associated with decreased projection and/or cephalic rotation of the tip.
- The major saddle nose corresponds to a marked lack of bony and cartilaginous support. This deformity requires a major reconstructive procedure like costal cartilage.

On the other hand, the injection of filler in the dorsum of the nose can correct minimal and moderate saddle nose. Non-surgical rhinoplasty can restore a harmonious appearance of the middle third of the nose and correct the defect in the dorsum. In moderate saddle nose, a columellar injection can be included, as a simple augmentation effect on the dorsum does not resolve the deficient support of the base of the nose [21]. The first examples of this approach were described more than one century ago. At that time, Syphilitic saddle nose was a common social stigma. In 1886, the scientific journal *Hospital* claimed that “the subcutaneous injection of liquid paraffin is the treatment of the saddle nose”. The immediate result was a “successful and effective correction of the defect” [22]. In 1907, Freeman reported some problems related to this technique. In particular, the spreading of the injected material was difficult to control, and many cases of embolism with blindness were described [23]. Many small case series of non-surgical treatment of saddle nose were published in more recent years and included treatment by injection of permanent and resorbable fillers [24,25]. The two biggest cases series of non-surgical rhinoplasty were published in 2010 and 2020. Rivkin and Soliemanzadeh published a retrospective, 4-year clinical review of 385 injections with calcium hydroxylapatite [26]. The cosmetic indications were maximal bridge height augmentation from the radix down the length of the dorsum in Asians and African Americans, camouflage of a dorsal hump, and correction of surgery in Caucasians, whereas Hispanic Americans generally presented for correction of a droopy nasal tip or a dorsal bump. Primary saddle nose is not reported as an indication. In 2020, Harb and Brewster retrospectively reviewed a big case series of 5000 injections of cross-linked hyaluronic acid gels in the nose [19]. The most common indication for treatment was dorsal hump (44%). Post-surgical correction was second most common (20%). Other indications were a drooping nasal tip, lack of definition, frontal asymmetry, and bulbous tip. The primary saddle nose is not described.

Significant case series of injections of fat graft for primary correction of the saddle nose are not reported in the scientific literature. On the opposite side, its use is successfully described in small case series of correction of post-rhinoplasty sequelae [27,28]. The safety and efficacy of different non-surgical rhinoplasty techniques is a fairly controversial matter. In particular, blindness is a devastating complication of injection of fillers because the dorsal nasal artery is a branch of the internal carotid artery. Irreversible blindness is linked to the retrograde flow of filler to the ophthalmic artery via the dorsal nasal artery [4]. Sorensen et al., specifically analyzing blindness caused by nose filler injection, reported 60 cases in the last 15 years, 89.9% of them after filler injection and 11.1% after injection of fat [29]. The most common injection location was the nose (*n* = 33, 55.0%), and the second was the glabella (*n* = 21, 35.0%) [29]. Albeit, only 0.09% (*n* = 8) of cases analyzed by DeVictor et al. reported vision loss in an overall of 8604 patients who underwent non-surgical rhinoplasty. This side effect undoubtedly implies a need for further optimization to increase the safety of the injection of fillers into the nose [30]. Injection by cannula appears to be a relatively safe procedure compared with needles. However, the use of a blunt-tip cannula still poses a potential risk. Previous studies have reported that ocular complications were induced by a 25G cannula and even by a 23G cannula. No complications were reported by using 22G cannulas or bigger ones [31].

Arteries run above the fibromuscular layer. For this reason, injection at the supraperiosteal layer is suggested as a safe injection plane [32]. However, irreversible blindness due to vascular occlusion could be partially prevented by artery detection before the injection. Lee et al. [33] showed that some arteries can be detected by Doppler ultrasound at the deep layer; thus, there was no completely safe layer for injection. Therefore, it is recommended that a Doppler ultrasound be performed before a nose injection. In this way, when the Doppler ultrasound probe is used at the midline of the nose, an abnormal position of the dorsal nasal artery can be detected, in particular, when it crosses the midline of the nose [34].

According to the previous literature, performing a gentle injection in the supraperiosteal layer with a 22G cannula (or bigger) with Doppler ultrasound guidance is relatively safe. In the authors’ experience, no major complications (visual loss, skin necrosis, etc.) were observed with this procedure. For this reason, they recommend the use of this technique for moderate saddle nose non-surgical rhinoplasty.

## Data Availability

Data are available upper reasonable request to authors.
